# Formation of orogenic gold deposits by progressive movement of a fault-fracture mesh through the upper crustal brittle-ductile transition zone

**DOI:** 10.1038/s41598-022-22393-9

**Published:** 2022-10-17

**Authors:** Miguel Tavares Nassif, Thomas Monecke, T. James Reynolds, Yvette D. Kuiper, Richard J. Goldfarb, Sandra Piazolo, Heather A. Lowers

**Affiliations:** 1 Department of Geology and Geological Engineering, Colorado School of Mines, Golden, CO 80401 USA; 2 FLUID INC., Denver, CO 80202 USA; 3 Institute of Geophysics and Tectonics, University of Leeds, Leeds, LS2 9JT UK; 4 U.S. Geological Survey, Denver, CO 80225 USA

**Keywords:** Petrology, Structural geology

## Abstract

Orogenic gold deposits are comprised of complex quartz vein arrays that form as a result of fluid flow along transcrustal fault zones in active orogenic belts. Mineral precipitation in these deposits occurs under variable pressure conditions, but a mechanism explaining how the pressure regimes evolve through time has not previously been proposed. Here we show that extensional quartz veins at the Garrcon deposit in the Abitibi greenstone belt of Canada preserve petrographic characteristics suggesting that the three recognized paragenetic stages formed within different pressure regimes. The first stage involved the growth of interlocking quartz grains competing for space in fractures held open by hydrothermal fluids at supralithostatic pressures. Subsequent fluid flow at fluctuating pressure conditions caused recrystallization of the vein quartz and the precipitation of sulfide minerals through wall-rock sulfidation, with some of the sulfide minerals containing microscopic gold. These pressure fluctuations between supralithostatic to near-hydrostatic conditions resulted in the post-entrapment modification of the fluid inclusion inventory of the quartz. Late fluid flow occurred at near-hydrostatic conditions and resulted in the formation of fluid inclusions that have not been affected by post-entrapment modification as pressure conditions never returned to supralithostatic conditions. This late fluid flow is interpreted to have formed the texturally late, coarse native gold that occurs along quartz grain boundaries and in open spaces. The systematic evolution of the pressure regimes in orogenic gold deposits such as Garrcon can be explained by relative movement of fault-fracture meshes across the base of the upper crustal brittle-ductile transition zone. We conclude that early vein quartz in orogenic deposits is precipitated at near-lithostatic conditions whereas the paragenetically late gold is introduced at distinctly lower pressure.

## Introduction

Orogenic gold deposits, which account for about one-third of the world’s gold production^1^, are comprised of complex quartz vein arrays that formed as a result of focused hydrothermal fluid flow along trans-crustal fault zones of mixed brittle-ductile character^2^. The ore-forming fluids are produced by crustal devolatilization during metamorphism in active orogens^3,4^. The formation of orogenic gold deposits is interpreted to occur within the upper crustal brittle-ductile transition zone, referred to as the continental seismogenic zone in young orogenic belts, that separates the lithostatically pressured lower crust from the overlying portion of the crust where near-hydrostatic pressures prevail^5–8^. Mineral deposition occurs as a consequence of fault-valve action along the controlling fault zone through fault failure, which permits episodic high-flux discharge of metamorphic fluids from the deeper-seated reservoir into upper crust^5–8^.

Although the structural framework of orogenic gold deposits is well studied, there is considerable uncertainty as to how large-scale fluid flow processes predicted by the fault-valve model translate to observations that can be made at the scale of individual veins or at microscopic scales. This knowledge gap, at least in part, arises from the fact that quartz in orogenic gold veins is typically recrystallized and primary textural relationships have been largely obliterated^9–13^. Dynamic recrystallization also results in widespread destruction of the primary fluid inclusion inventory in the quartz^9–13^. In addition, most fluid inclusions have been affected by post-entrapment modification^10,12–18^ caused by pressure changes that occurred during and after metal precipitation^12–14^. As a result, the pressure and temperature conditions of quartz deposition and gold precipitation cannot be accurately reconstructed in most orogenic gold deposits.

Here we report on the occurrence of extensional quartz veins from the Garrcon orogenic gold deposit in the Neoarchean Abitibi greenstone belt of Canada that show exceptionally well-preserved primary textures, although the related fluid inclusion inventory has been severely affected by post-entrapment modification. It is demonstrated that the textural evidence alone allows the reconstruction of the mechanisms of quartz vein formation and the relative timing of gold introduction, constraining the temporal evolution of the fluid flow regime at the scale of individual veins. A framework is proposed for how orogenic gold deposits can form through progressive movement of a network of interlinked shear fractures and extensional fractures—so-called fault-fracture meshes^19^—across permeability barriers within the upper crustal brittle-ductile transition zone, which is a complex region of alternating brittle and ductile behavior^20^.

## Geological setting

With a total endowment of more than ~ 200 million ounces of gold, the southern Abitibi greenstone belt of Ontario and Quebec is one of the most prolific orogenic gold provinces in the world^21,22^. The belt encompasses Neoarchean metavolcanic rocks formed in a submarine setting between 2795 and 2695 Ma^22–24^, and flysch-like metasedimentary rocks of the Porcupine assemblage deposited as a result of crustal thickening and emergence of a shallow marine or subaerial hinterland^22,25^ between 2690 and 2685 Ma^22–24^. Large-scale folding and thrusting during a deformational event occurring prior to 2679 Ma^22,25^ resulted in the development of a regional terrestrial unconformity surface that is overlain by 2679–2669 Ma^22–24^ molasse-like metasedimentary rocks of the Timiskaming assemblage, which locally contain intercalated subaerial alkaline metavolcanic units^22,26–28^.

**Figure 1:**
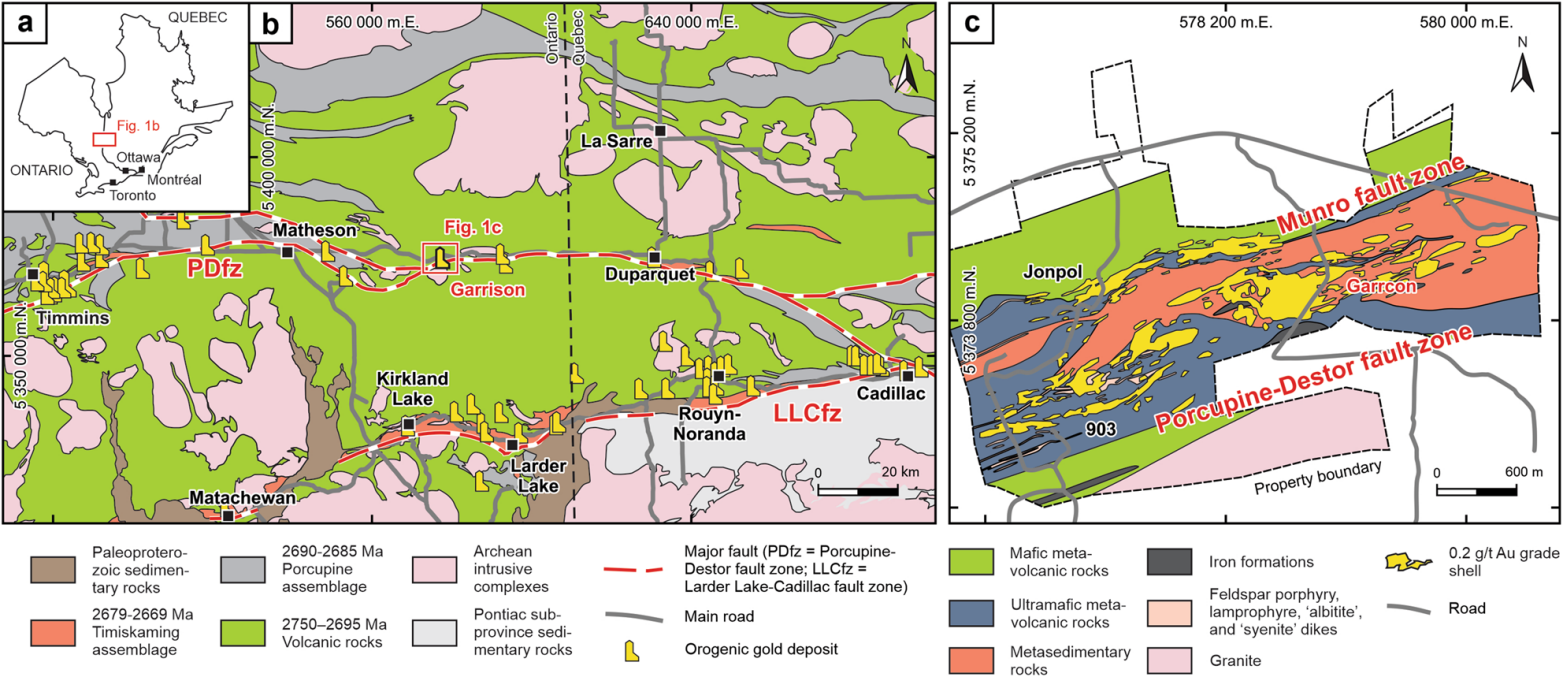
Geological setting of the Garrcon deposit. (**a**) Location in the Superior Province. (**b**) Map of the southern Abitibi greenstone belt showing major fault zones and locations of orogenic gold deposits along the Porcupine-Destor and Larder Lake-Cadillac fault zones. Map modified from Ref.^22^. (**c**) Geology of the Garrison gold camp and the Garrcon deposit. Map modified from Ref.^31^. Figure was created using CorelDRAW^®^2021 (https://www.coreldraw.com/).

Crustal shortening and thick-skinned thrusting resulted in the structural burial of the molasse-like metasedimentary rocks after 2669 Ma^22,25^. Panels of the metasedimentary rocks of the Timiskaming assemblage are preserved in the footwall of these thrusts, which are today represented by major fault zones transecting the supracrustal rocks of the southern Abitibi greenstone belt. All major gold camps in the belt are located along these crustal-scale fault zones^21,22,25,29^, which include the E-trending Porcupine-Destor fault zone in the north and the Larder Lake-Cadillac fault zone in the south (Fig. 1a). The metavolcanic and metasedimentary host rocks of the orogenic gold deposits in the southern Abitibi greenstone belt were metamorphosed to prehnite-pumpellyite to lower greenschist facies prior to ore formation^22,30^.

**Figure 2:**
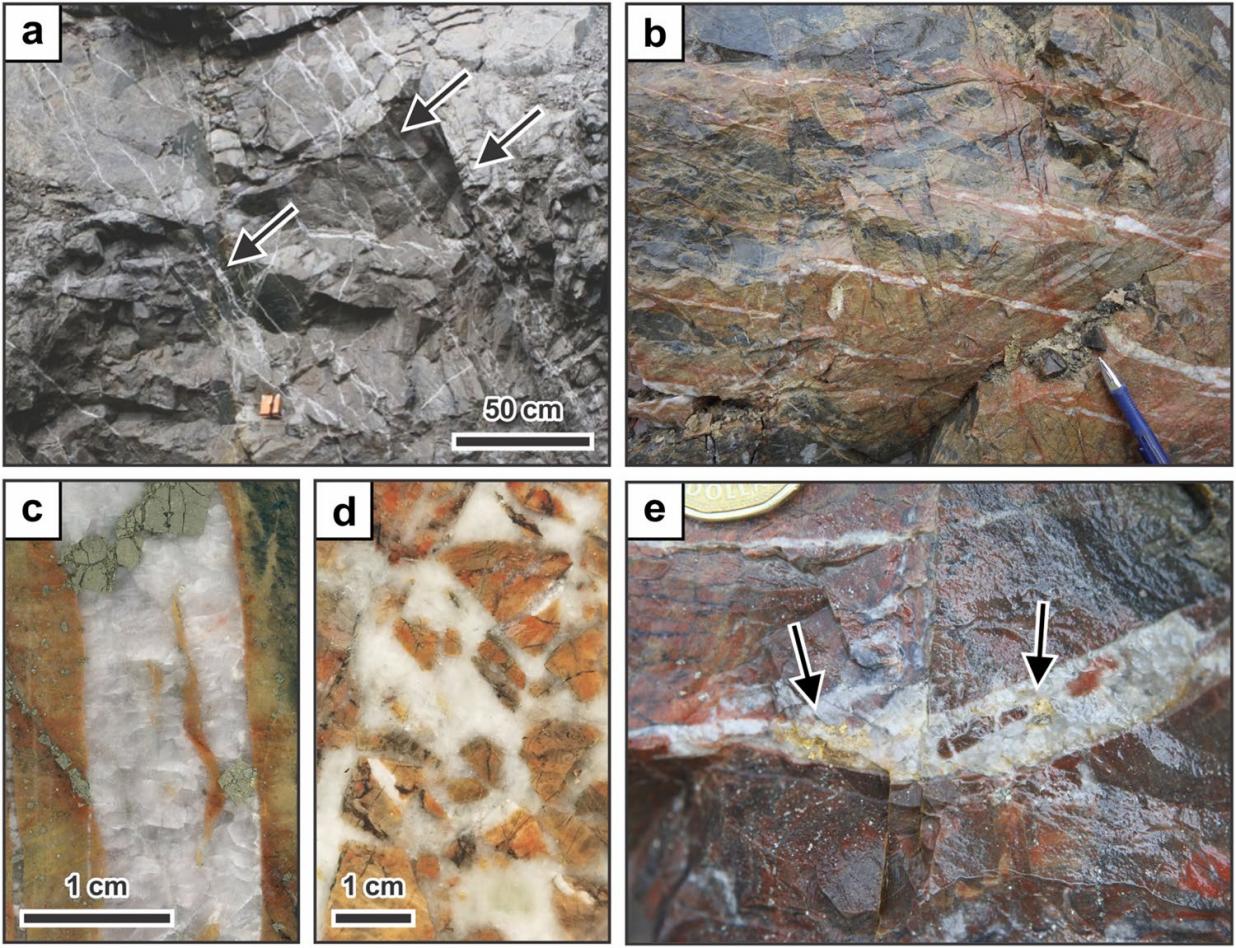
Characteristics of quartz veins. (**a**) E-dipping quartz veins (arrows) crosscutting metasedimentary rocks. (**b**) Extensional veins surrounded by halos of intense albite alteration. (**c**) Hand specimen of extensional quartz vein containing pyrite. The metasedimentary rock surrounding the vein is albite-altered. (**d**) Hand specimen of hydrothermal breccia cemented by quartz. The clasts of wall-rock in the breccia are intensely albite-altered. (**e**) Extensional vein containing abundant visible gold (arrows). The visible gold appears to be paragenetically late and crosscuts the quartz forming the vein.

The Garrcon gold deposit (20.6 million tonnes of ore containing 636,000 oz Au^31^) is located in the Garrison camp near the provincial border between Ontario and Quebec within a transtensional segment of the Porcupine-Destor fault zone (Fig. 1b). The deposit occurs within a ~ 600-m-wide fault block that is bound by the subvertical Munro fault zone in the north and the Porcupine-Destor fault zone in the south^32^. The ore zones consist of east-dipping sets of veins (Fig. 2a) that are hosted by massive metagreywacke of the Timiskaming assemblage and meta-intrusive rocks^32^. Abundant extensional veins (Fig. 2b) are ~ 1 mm to ~ 5 cm in width (Fig. 2c) and are locally associated with minor zones of hydrothermal brecciation (Fig. 2d). The quartz veins are surrounded by distinct beige-colored halos caused by pervasive albite alteration of the metasedimentary and meta-intrusive host rocks (Fig. 2b–d). The altered wall-rocks contain abundant disseminated pyrite. Visible gold in the extensional veins appears to be paragenetically late and commonly is present in fractures cutting the quartz (Fig. 2e).

## Vein quartz petrography

**Figure 3:**
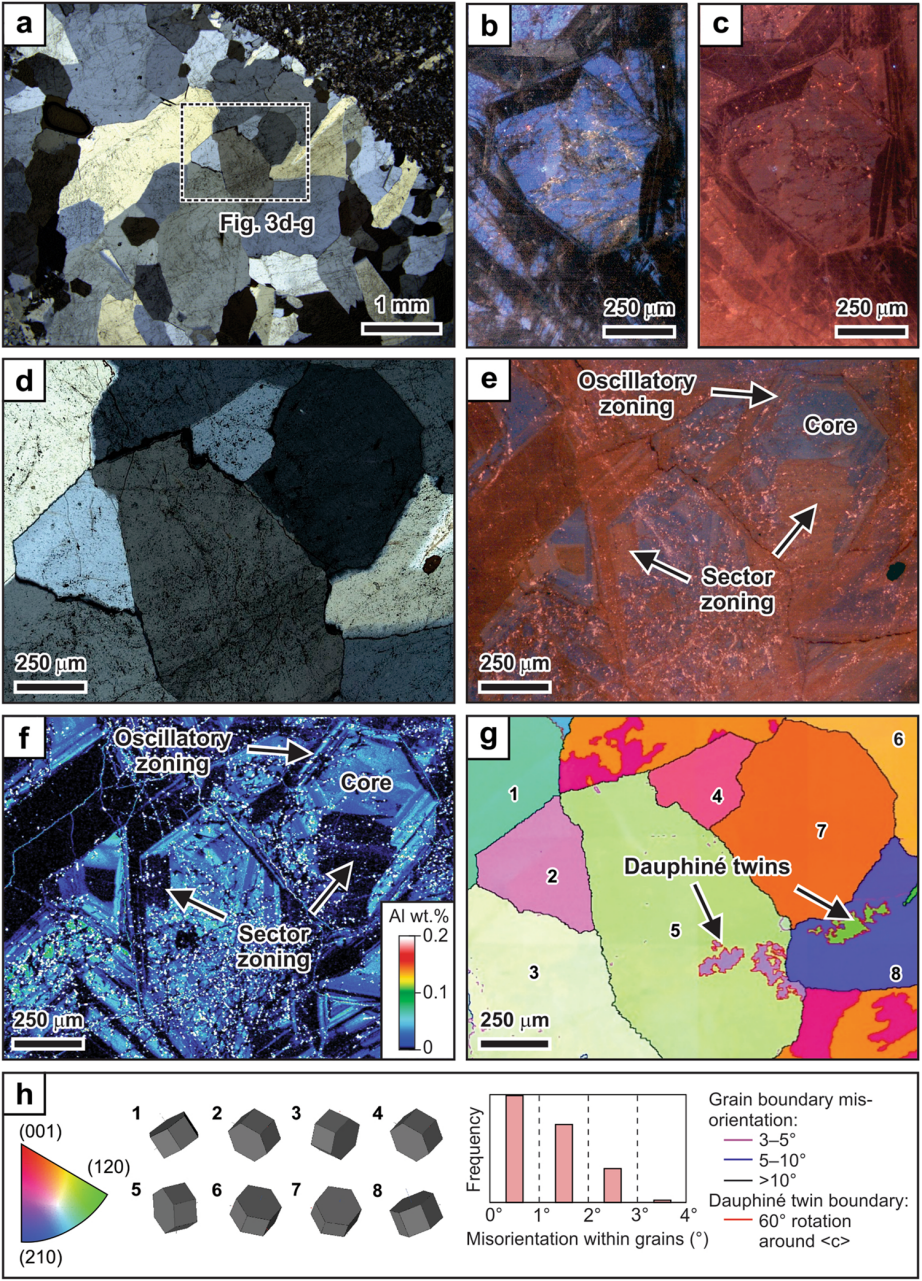
Petrographic characteristics of extensional vein quartz. (**a**) Vein consisting of elongate to blocky grains with planar or interlocking grain boundaries. (**b**) Cathodoluminescence image showing that the quartz grains have a short-lived (~ 15 s) blue emission. Quartz surrounding clusters of fluid inclusions affected by post-entrapment modification shows a yellow emission. (**c**) Long-lived (~ 100 s) red-brown to brown cathodoluminescence of quartz grains showing primary growth zoning. (**d**) Crossed-polarized light image of elongate and blocky quartz. (**e**) Corresponding long-lived (~ 300 s) cathodoluminescence image showing oscillatory and sector zoning of the grains. (**f**) Electron microprobe map of Al illustrating that the growth zones in the quartz grains observed by cathodoluminescence imaging are compositionally distinct. (**g**) Electron backscatter diffraction orientation map of elongate to blocky quartz grains. Grain orientations are represented by the different colors. Grain boundaries are color-coded depending on the grain boundary misorientation. Boundaries of Dauphiné twins are represented by the red lines. (**h**) Results of the electron backscatter diffraction analysis. The diagram on the left shows the color scheme used for the grain orientations relative to main crystal axis perpendicular to the map plane. The diagram in the middle shows a 3D representation of grain orientations of the numbered grains in (**g**). The histogram depicts the relative frequency of grains plotted as function of the changes in crystallographic orientation within individual grains. The internal misorientation does not exceed four degrees across individual grains.

Thin section petrography shows that key primary textural relationships are preserved in samples of the extensional veins from Garrcon. The veins consist mostly of elongate to blocky quartz grains that commonly increase in grain size from the vein walls toward the vein centers (Fig. 3). The quartz grains are approximately perpendicular to the wall-rock contact along the vein margins but show no preferred shape or crystallographic orientation in the vein centers (Fig. 3a). Grain boundaries between adjacent quartz grains range from planar to interlocking (Fig. 3a). Optical cathodoluminescence (CL) imaging reveals that the quartz grains have a short-lived blue emission (Fig. 3b) that changes to light purple during continued electron bombardment. The quartz exhibits a long-lived red-brown to brown CL color (Fig. 3c). The elongate and blocky quartz crystals appear unzoned in plane and cross-polarized light (Fig. 3d) but exhibit complex oscillatory and sector zoning patterns in CL (Fig. 3e,f).

Electron microprobe mapping shows that the growth zones in the elongate to blocky quartz grains recognized by CL vary in Al content. Growth zones showing a bright luminescence have comparably high Al concentrations (Fig. 3e,f). Electron backscatter diffraction analysis illustrates that the elongate to blocky quartz grains in the vein centers have different orientations, but that the crystallographic orientation within individual grains does vary by more than four degrees. The lack of crystallographic orientation changes within individual grains (Fig. 3g,h) suggests that stress-induced dislocation glide or creep was minor after quartz crystal growth within the veins. Mechanical Dauphiné twins are present only in some quartz grains (Fig. 3g,h). The quantitative orientation analysis confirms that the complex oscillatory and sector zoning visible in CL is a primary characteristic of the elongate to blocky quartz grains.

**Figure 4:**
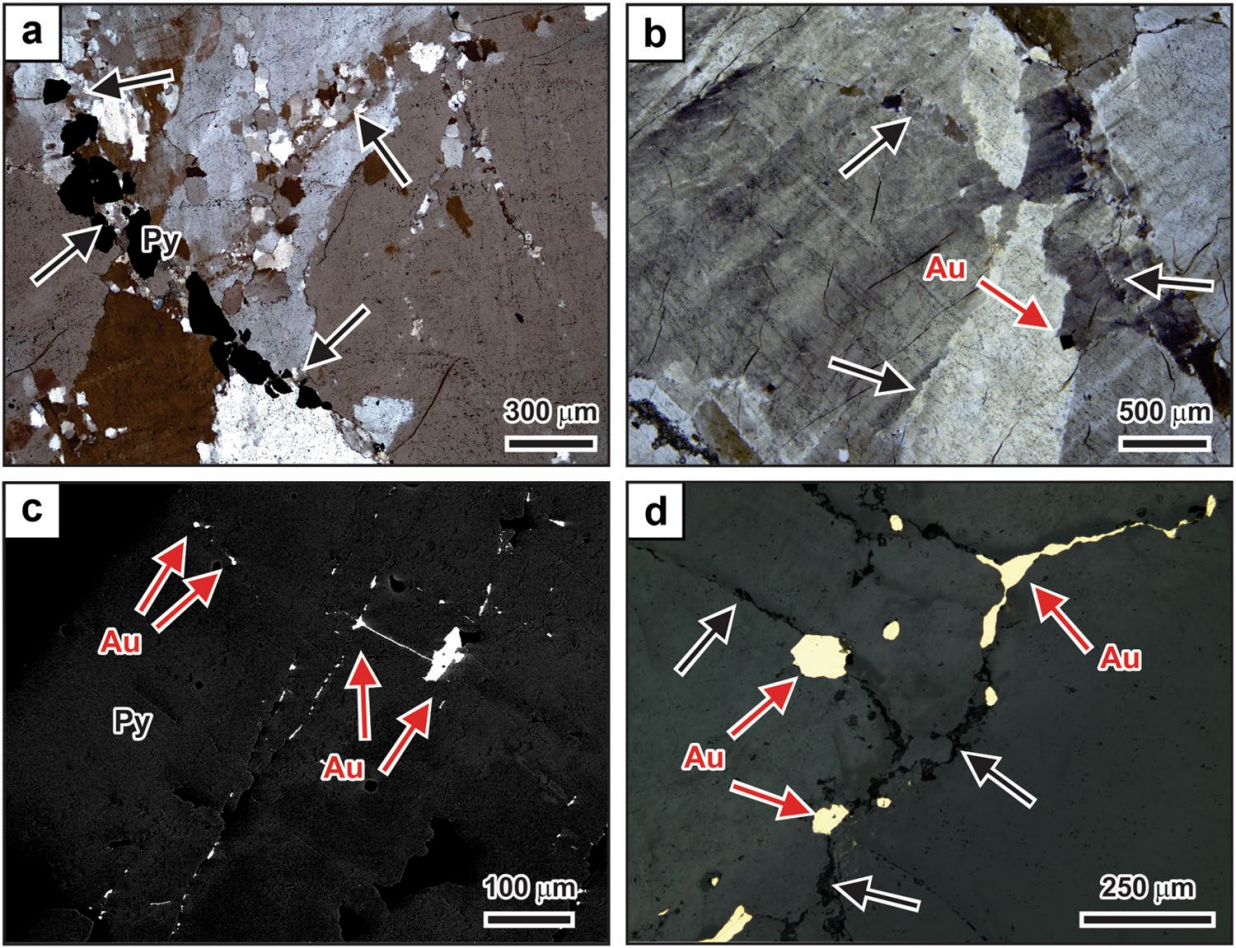
Petrographic characteristics illustrating paragenetic relationships. (**a**) Ribbons of anhedral pyrite and polycrystalline quartz aggregates (arrows) crosscutting originally elongate to blocky quartz grains. Crossed-polarized light. (**b**) Narrow bands of small polycrystalline quartz grains (arrows). The bands transect originally elongate to blocky grains or occur along original grain boundaries. Gold occurs at a grain boundary (red arrow). Crossed-polarized light. (**c**) Backscatter electron image of pyrite grain exhibiting patchy compositional zoning. Micron-sized gold occurs as inclusions in the pyrite and along microfractures transecting the pyrite (red arrows). Subtle patchy zoning in the pyrite is caused by variations in the As content. (**d**) Late gold (red arrows) located along quartz grain boundaries (arrows) in reflected light. Au = gold; Py = pyrite.

However, in many vein samples from Garrcon, the originally elongate and blocky quartz grains are affected by recrystallization (Fig. 4). Recrystallization is particularly pronounced surrounding microscopic ribbons of pyrite and minor arsenopyrite that cut across the vein quartz or are present along grain boundaries between larger quartz grains. Polycrystalline aggregates consisting of small (10‒20 μm) and nearly equidimensional grains occur in these zones of recrystallization (Fig. 4a,b). The spatial association between recrystallized quartz and sulfide minerals suggests that the sulfides formed paragenetically after the growth of the early elongate and blocky quartz grains, contemporaneous with recrystallization of the earlier quartz. Pyrite occurring in the sulfide ribbons shows complex patchy zoning patterns in backscatter electron images that are primarily related to variations in As content (Fig. 4c). Gold occurs as microscopic inclusions in the pyrite. The gold inclusions are encapsulated by the pyrite or, more commonly, occur along small fractures within the pyrite grains (Fig. 4c).

Coarse native gold is texturally late and present within microfractures transecting earlier quartz grains or at grain boundaries of the vein quartz (Fig. 4b,d). Native gold is also present in late vugs filled with clear euhedral quartz crystals or calcite and is commonly closely associated with chlorite. Grains of native gold are present along healed microfractures cutting the latest quartz and calcite.

## Fluid inclusion petrography

**Figure 5:**
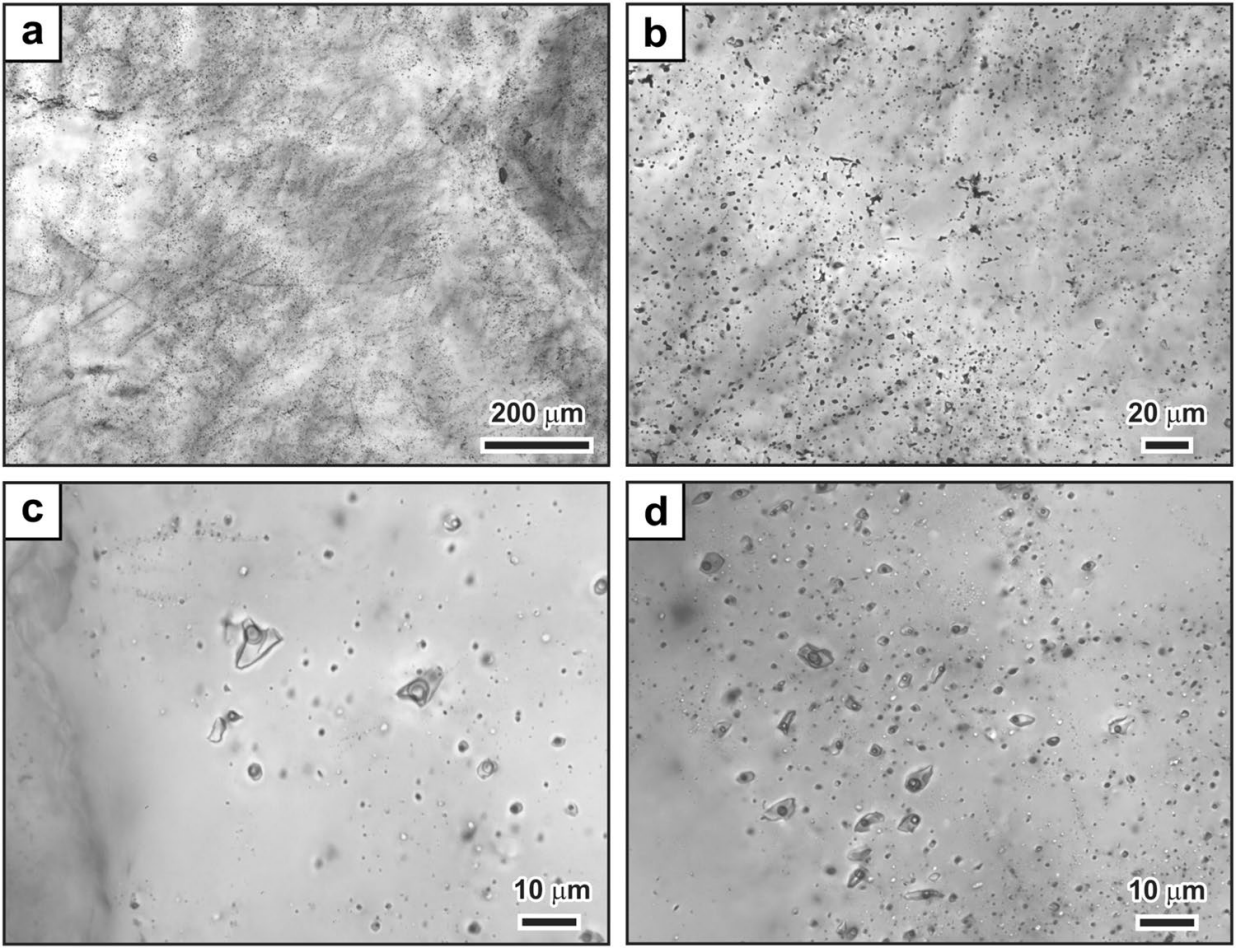
Fluid inclusion characteristics of extensional vein quartz. (**a**) Wispy texture from myriads of healed microfractures defined by inclusions that are typically < 5 μm in size. (**b**) High magnification image showing decrepitation textures and inclusions with variable phase proportions. (**c**) Healed microfracture of equant-shaped H_2_O–CO_2_ fluid inclusions. The inclusions contain aqueous liquid and a double bubble composed of a bubble of carbonic liquid that encloses a bubble of carbonic vapor. The inclusions show consistent volumetric proportions within the single inclusion assemblage. (**d**) Quartz crystal containing H_2_O-dominant inclusion assemblages with consistent volumetric proportions of liquid and vapor. Such assemblages of inclusions predominate in the latest, clearest euhedral quartz.

**Figure 6:**
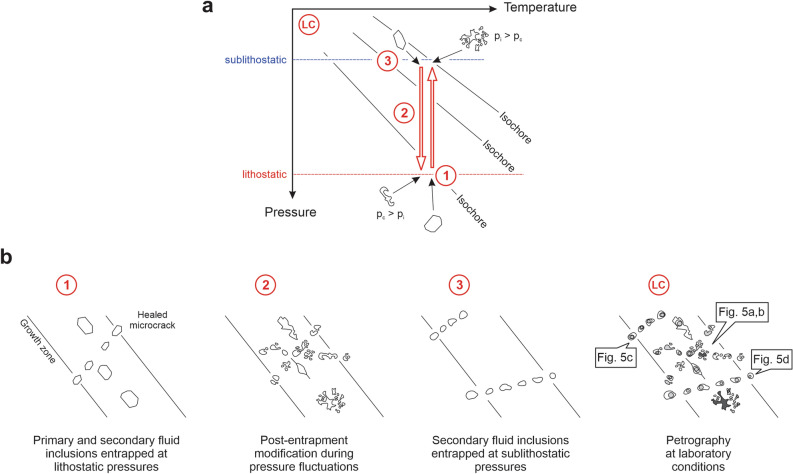
Diagram showing how the fluid inclusion petrography of vein quartz from the Garrcon deposit was formed in different pressure regimes. (**a**) Pressure–temperature diagram showing the conditions at which fluid inclusion assemblages in the veins are entrapped or observed. Early quartz contains primary and secondary fluid inclusions entrapped at lithostatic conditions (1). Post-entrapment modification of the fluid inclusions occurs during pressure fluctuations between lithostatic and sublithostatic conditions (2), as the fluid inclusions are not strong enough to withstand the pressure differentials from entrapment followed by a pressure drop (internal pressure > confining pressure) or entrapment followed by pressure increases (confining pressure > internal pressure). Secondary fluid inclusion assemblages are entrapped at hydrostatic conditions (3). Study of the fluid inclusion petrography occurs at laboratory conditions (LC). (**b**) Petrographic characteristics of fluid inclusion assemblages entrapped at the different pressure and temperature conditions. Early quartz may contain primary fluid inclusions along growth zones and secondary fluid inclusions along healed microcracks (1). The early formed fluid inclusions are affected by post-entrapment modification during pressure fluctuations (2). New secondary fluid inclusion assemblages are also formed at this stage and modified during subsequent pressure changes. Late secondary fluid inclusions are entrapped at sublithostatic pressures (3). At laboratory conditions (LC), a complicated integrated history of different fluid inclusion assemblages formed at different pressure and temperature conditions through time can be observed. Inclusions rich in CO_2_ can display double bubbles at room temperature, whereas H_2_O-dominant fluid inclusions may consist of a liquid and vapor phase only. Fluid inclusion assemblages that show consistent volumetric proportions among the various phases within individual inclusions must have been entrapped at sublithostatic pressures and never have been subjected to lithostatic conditions. *LC* laboratory conditions, *p*_*c*_ confining pressure, *p*_*i*_ internal pressure.

The early elongate to blocky quartz and recrystallized quartz grains are cloudy in thin section due to abundant secondary fluid inclusions forming dense, wispy arrays (Fig. 5a). Decrepitation textures are common (Fig. 5b), indicating that many fluid inclusions hosted by the vein quartz were affected by post-entrapment modification (Fig. 6). However, some secondary inclusion assemblages do not show evidence of post-entrapment modification, but instead the inclusions contain consistent phase proportions (Fig. 5c,d). Such assemblages are discernable in clearer quartz locally present in the veins. These assemblages of fluid inclusions containing consistent phase proportions include three-phase fluid inclusions with double bubbles (Fig. 5c), in which an aqueous liquid wets the inclusion walls and suspends a bubble of carbonic liquid that encloses a bubble of carbonic vapor, as well as two-phase H_2_O-dominant fluid inclusions (Fig. 5d).

## Discussion

The vein textures at Garrcon record the evolution of the hydrothermal system that formed this orogenic gold deposit. The paragenetically earliest event resulted in the deposition of the barren, elongate to blocky quartz in the extensional veins. This was followed by the formation of microscopic sulfide ribbons along many of the ubiquitous microfractures cutting the earlier quartz and along grain boundaries between the elongate and blocky quartz grains, with the earlier quartz recrystallizing along these microscale zones of fluid flow. Pyrite formed during this paragenetic stage contains gold as microscopic inclusions. Later, formation of euhedral quartz in open spaces occurred, which was accompanied by late chlorite growth. Paragenetically latest is native gold, which occurs along microfractures cutting late euhedral quartz and calcite, or along grain boundaries of the earlier elongate and blocky quartz.

**Figure 7:**
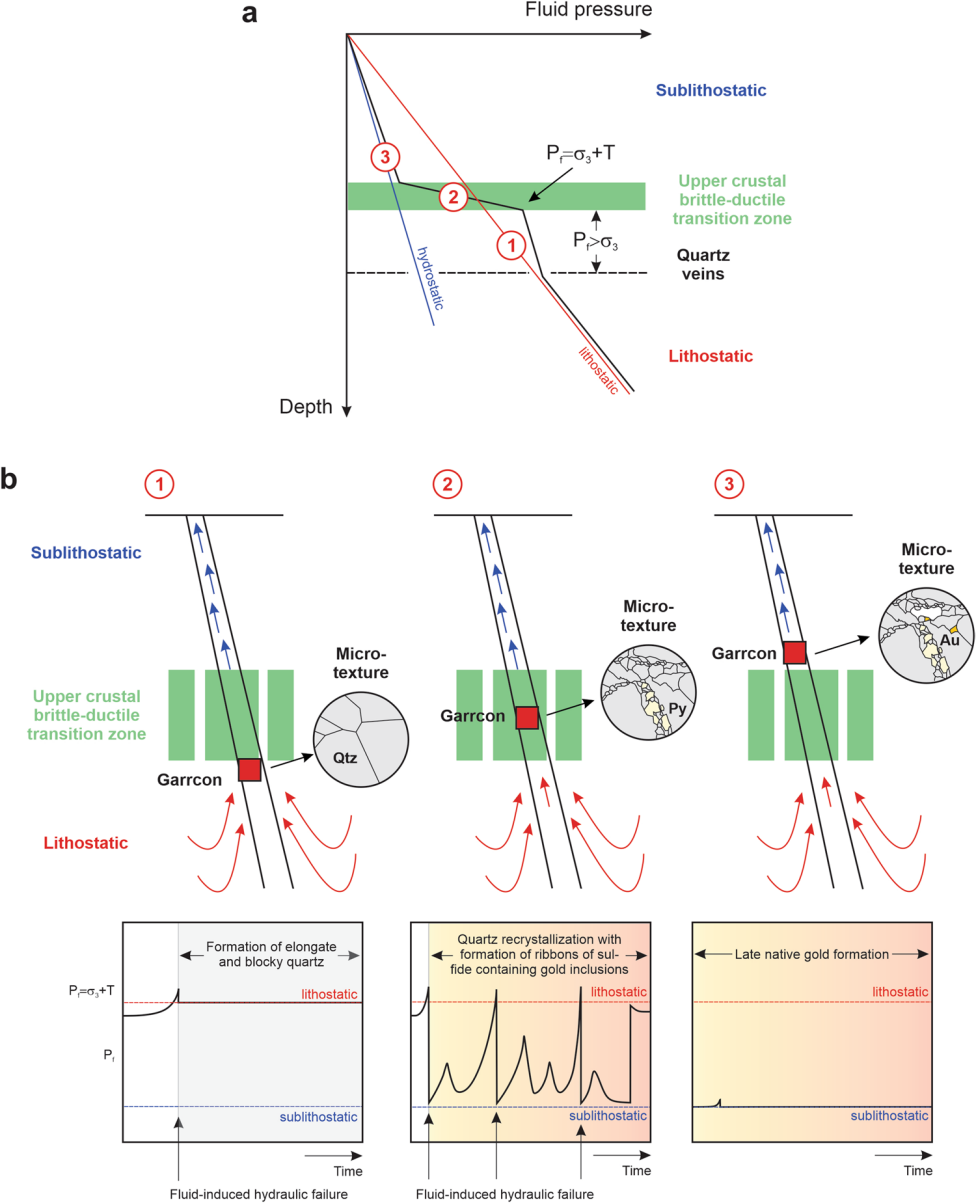
Diagram depicting the evolution of the hydrothermal system forming the Garrcon deposit. (**a**) Hypothetical profile of fluid pressure within a crustal fault zone transecting a permeability seal within the upper crustal brittle-ductile transition zone. The lower part of the crust is ductile and lithostatic pressures prevail. The upper part of the crust is brittle allowing fluid flow at near-hydrostatic conditions. The diagram shows the environments in which the three paragenetic stages identified at Garrcon are likely to have formed (1 = Formation of elongate and blocky quartz; 2 = Quartz recrystallization concomitant with the formation of sulfide ribbons containing gold inclusions; 3 = Late native gold formation). However, note that the diagram does not account for changes in pressure regime over time, which are required to explain the observation that all three paragenetic stages occur in single vein samples. Modified from Ref.^8^. (**b**) Exhumation model explaining the progressive changes in fluid pressure regimes over time. For each paragenetic stage, the red box denotes the relative position of the Garrcon fault-fracture mesh with respect to the upper crustal brittle-ductile transition zone. Elongate and blocky quartz forms early below the low-permeability seal in lithostatically pressured rocks (1). Crack initiation occurs as the fluid pressure P_f_ exceeds σ_3_ + T, with T being the tensile strength of the rock. Lithostatic pressures are required to keep the fractures open enough allowing growth of quartz crystals competing for space. Quartz recrystallization and formation of sulfide ribbons containing gold inclusions (2) takes place at fluctuating pressures as the location of the fault-fracture mesh at Garrcon relative to the permeability seal has changed. Pressure cycling promotes fluid-rock interaction and sulfidation of the wall-rocks, resulting in the formation of sulfides with microscopic inclusions of gold. Late native gold formation (3) in open spaces occurs at sublithostatic conditions as the fluids escape into the upper crust. The three pore fluid versus time diagrams in the lower part of the image are modified from Ref.^33^. Au = gold; Py = pyrite; Qtz = quartz.

The formation of the extensional quartz veins at Garrcon can be related to episodic fluid flow through a fault-fracture mesh located in a fault block between the subvertical Munro and Porcupine-Destor fault zones^32^. These crustal-scale fault zones allowed upflow of geopressured fluids created by metamorphic devolatilization deeper in the crust^2–4^. Fluid pressure build-up beneath a permeability barrier within the upper crustal brittle-ductile transition zone (Fig. 7) resulted in intermittent, catastrophic hydraulic fracturing^7,8^. Following failure, the fractures created were held open by the supralithostatically overpressured fluids until they were sealed by quartz^33,34^. The comparably large volume of flow resulted in significant quartz deposition^35^ and initial vein formation through cooling of the metamorphic fluids when migrating through the fracture mesh away from the main faults controlling upflow from depth. Continuous quartz precipitation in the newly formed, gaping fractures explains the interlocking texture of the paragenetically early quartz grains that competed for space, with each vein being sealed during a single episode of fluid flow^33,36–38^. The rate of separation of the vein walls caused by the high-pressure fluids must have been faster than the rate of mineral deposition to avoid formation of crack-seal textures^39^.

At Garrcon and other orogenic deposits^11–14^, recrystallization of the early quartz occurred concomitantly with sulfide formation as a result of fluid advection through the earlier formed veins (Fig. 7). Local failure in the presence of hydrothermal fluids caused the dynamic recrystallization of the earlier quartz^40^ and resulted in the observed textural association between the fine-grained polycrystalline quartz aggregates and the pyrite and arsenopyrite forming microscopic ribbons that cut the elongate and blocky quartz or are located along pre-existing quartz grain boundaries. In many vein samples, multiple crosscutting sulfide ribbons are present suggesting that individual veins have reopened multiple times causing repeated dissolution and recrystallization of the vein quartz.

The petrography of the fluid inclusions in the quartz (Fig. 6) provides strong evidence for the hypothesis that the formation of the sulfide ribbons and associated recrystallization of the earlier quartz was associated with large fluctuations in fluid pressure. Fluid inclusions entrapped during the formation of the early elongate and blocky quartz in the gaping structures at supralithostatic pressures have been affected by post-entrapment textural modification (Fig. 6). Post-entrapment modification takes place when large pressure differentials occur between fluid inclusions and their surroundings^11–14^, which is the case during decompression from supralithostatic to near-hydrostatic fluid pressures. However, the early elongate and blocky quartz as well as the recrystallized quartz also contain myriads of healed microfractures defined by secondary fluid inclusions that were potentially entrapped at near-hydrostatic pressures and subsequently modified during pressure build-up to lithostatic conditions (Fig. 6), suggestive of pressure cycling. Due to the post-entrapment modification of the fluid inclusions^14,41,42^, fluid inclusion microthermometry cannot be used to determine the pressure and temperature conditions^41,42^ of formation of the elongate and blocky quartz in the extensional veins at Garrcon, or the subsequent fluid-mediated recrystallization.

Based on the vein petrography and fluid inclusion petrographic evidence, the occurrence of abundant pyrite and minor arsenopyrite in ribbons cutting the earlier elongate and blocky quartz and the presence of microscopic gold in the pyrite must thus be linked to cyclic variations in the pressure regime. During pressure build-up, hydrothermal fluids are injected into the wall-rock surrounding fluid conduits. Sulfide formation occurs around the veins due to wall-rock sulfidation, resulting in a decrease in the amount of reduced sulfur in solution^2,4^. Sulfide deposition within the veins takes place as hydrothermal fluids that have reacted with the wall-rock are driven back into the vein during reversal in hydraulic head associated with the opening of the veins^37^. Gold transported by sulfide complexes is deposited^2,4^ together with the sulfide minerals formed through wall-rock sulfidation in the wall rocks and within the veins. Gold forms microscopic inclusions in pyrite or occurs along microfractures cutting across pyrite grains formed during an earlier cycle of fluctuating pressures.

The cyclic fluctuations in fluid pressure can be explained by temporary drainage of the geopressured fluids from the permeability barrier into the overlying near-hydrostatic realm (Fig. 7). Prior to failure, the fluid pressure builds up to supralithostatic conditions within and below the permeability barrier. During fault activation, the pressure drops to near-hydrostatic conditions and the hydrothermal fluids drain. As the fluids are drained and fractures providing throughgoing permeability are sealed, the pressure in the fluid conduit returned to near-lithostatic conditions.

Textural observations at Garrcon (Fig. 4d) suggest that deposition of native gold along microfractures, grain boundaries, and in open spaces was late in the paragenesis. Similar to other orogenic gold deposits^12–14^, late gold introduction at Garrcon occurred subsequent to the permanent decompression of the fault-fracture mesh to near-hydrostatic conditions (Fig. 7). Secondary fluid inclusion assemblages present in recrystallized quartz, in clear quartz overgrowths with euhedral crystal terminations, or in euhedral quartz or calcite grown in open spaces have not been affected by post-entrapment modification (Fig. 6). Although these fluid inclusion assemblages show consistent liquid to vapor volumetric proportions, homogenization temperatures for these inclusions were not determined because they would only yield minimum temperatures. No evidence for phase immiscibility existed within the fluid inclusion assemblages, nor are there independent constraints on the pressures that prevailed at the time of gold deposition at Garrcon. Nevertheless, the petrography of these late inclusion assemblages showing consistent phase proportions provides unequivocal evidence that high pressure conditions were never reestablished during or after the formation of the clear quartz, after which native gold was deposited.

Based on the findings of this study, it is hypothesized here that the late gold precipitation within the fault-fracture mesh at Garrcon occurred because of the pressure drop metamorphic fluids experienced as they traversed the upper crustal brittle-ductile transition zone, escaping from the geopressured regime prevailing under ductile and mixed brittle-ductile conditions into the overlying, near-hydrostatically pressured, brittle crust (Fig. 7). This pressure drop may have caused direct native gold deposition or perhaps triggered the formation of gold colloids in the hydrothermal fluids^43–45^. The origin of the paragenetically late gold is unknown in the case of Garrcon. Previous workers studying other orogenic gold deposits suggested that the gold might have been derived from remobilization of gold that was originally deposited with pyrite and arsenopyrite earlier in the paragenesis^46–48^. There is no petrographic evidence for this process at Garrcon although it cannot be ruled out that gold remobilization occurred at greater depth, outside of the current deposit.

Systematic changes in pressure regime during vein formation at Garrcon, as indicated by mineral and fluid inclusion petrography, suggest that the structural setting governing fluid flow changed over time. It is hypothesized here that the fault-fracture mesh progressively moved across the upper crustal brittle-ductile transition zone over time (Fig. 7). This could have been accomplished through downward cooling of the metamorphic belt allowing the brittle-ductile transition to migrate down towards the core of the cooling orogen^49^ and/or regional uplift and exhumation^50,51^. Initial quartz vein formation occurred below a low-permeability barrier capping the geopressured portion of the crust. Fluid-induced recrystallization of the quartz and concomitant sulfide formation caused by the sulfidation of the wall-rocks occurred during intermittent breaching of this seal and drainage of the hydrothermal fluids into the overlying near-hydrostatically pressured portion of the crust. High pressures were never reestablished after deposition of the late clear quartz and the calcite as well as the late native gold deposition, which occurred along grain boundaries and in open spaces at near-hydrostatic pressure conditions.

## Conclusions

The mineral paragenesis observed in the extensional quartz veins at Garrcon as well as the petrographic characteristics of the fluid inclusion inventory of the vein quartz—which includes early fluid inclusions affected by post-entrapment modification and later unmodified fluid inclusions—are similar to those recently recorded in other orogenic deposits^12–14^. This suggests that the origins of orogenic gold deposits can be explained by a common mechanism of progressive movement of fault-fracture meshes across permeability barriers within the upper crustal brittle-ductile transition zone. This new model has important implications with regard to exploration strategy and grade control. The textural observations indicating that gold precipitates during two different distinct paragenetic stages could explain why grade distribution in these deposits is variable. Wall-rock sulfidation during pressure cycling between supralithostatic and near-hydrostatic conditions causes the deposition of early microscopic gold within sulfide minerals. Bonanza-type gold may occur in ore shoots where near-hydrostatic conditions were permanently established late in the paragenesis.

## Methods

Polished thin (~ 30 µm) and thick (~ 60 µm) sections of variably mineralized quartz veins from Garrcon were studied by optical petrography. Subsequent optical cathodoluminescence microscopy was conducted using a HC5-LM microscope by Lumic Special Microscopes, Germany. The microscope was operated at 14 kV and a current density of ~ 10 mA mm^−2^. Images were captured using a high-sensitivity, double-stage Peltier cooled Kappa DX40C CCD camera. Small-scale textural relationships were studied by scanning electron microscopy using a TESCAN Mira 3 LHM Schottky field-emission-scanning electron microscope with an attached Bruker XFlash 6|30 silicon drift detector for energy-dispersive X-ray spectroscopy. The trace element distributions of Al and Ti in selected quartz crystals were mapped using a JEOL JXA-8900 electron microprobe following the procedure of Ref.^52^. An accelerating voltage of 20 kV and a beam current of 100 nA (measured on the Faraday cup) were employed. At a detection limit of 105 ppm, the Al distribution map yielded useful information on compositional zoning of the quartz. The concentration of Ti in the quartz was typically below the detection limit of 300 ppm. Representative vein quartz samples were also studied by electron backscatter diffraction analysis using a FEI Quanta 450 field emission scanning electron microscope operated at 20 kV and low vacuum. Electron backscatter diffraction patterns were acquired with an EDAX Digiview IV detector set to 4 × 4 binning. Post-acquisition data processing was performed using the Oxford Instruments software suite.

## Data Availability

All data generated or analyzed during this study are included in this published article.
